# The determinants of caregiver use and its costs for elderly inpatients in Korea: a study applying Andersen’s behavioral model of health care utilization and replacement cost method

**DOI:** 10.1186/s12913-021-06677-w

**Published:** 2021-07-01

**Authors:** Jennifer Ivy Kim, Sukil Kim

**Affiliations:** 1 Department of Public Health, Graduate School, The Catholic University of Korea, 222- Banpo-daero, Seocho-gu, 06591 Seoul, Korea; 2 Department of Preventive Medicine, College of Medicine, The Catholic University of Korea, 222-Banpo-daero, Seocho-gu, 06591 Seoul, Korea

**Keywords:** Health expenditure, Elderly patient, Caregiving, Informal caregiver, Inpatient health care utilization, Andersen’s behavioral model

## Abstract

**Objectives:**

The average annual healthcare expenditure among elderly patients in Korea is increasing rapidly in indirect healthcare sectors, requiring an understanding of factors related to the use of both formal and informal caregivers. This study analyzed the characteristics of caregiver use and caregiving costs among elderly patients hospitalized due to acute illness or exacerbation of chronic diseases.

**Methods:**

A total of 819 study participants were selected from the 2017 Korea Health Panel Study Data. Replacement costing methods were applied to estimate the hours of informal caregiver assistance received by elderly inpatients. Elderly inpatients’ predisposing, enabling, and need factors were studied to identify the relationship between caregiver uses, based on Andersen’s behavior model. A two-part model was applied to analyze the factors related to care receipt and to estimate the incremental costs of care.

**Results:**

Elderly inpatients who used tertiary hospitals (OR: 2.77, p-value < 0.00) and received financial support (OR: 2.68, *p*-value < 0.00) were more likely to receive support from a caregiver. However, elderly inpatients living alone were lesser to do so (OR: 0.49, *p*-value < 0.00). Elderly inpatients with Medicaid insurance (β:0.54, *p*-value = 0.02) or financial aid (β: 0.64, *p*-value < 0.00) had a statistically positive association with spending more on caregiving costs. Additionally, financial support receivers had incremental costs of $627 in caregiving costs than nonreceivers.

**Conclusions:**

This study presented significant socioenvironmental characteristics of formal and informal caregiver use and the related expenditures. Healthcare management plans that encompass multiple social levels should be implemented to ease the caregiver burden.

**Trial registration:**

Retrospectively registered.

## Background

In 2019, the number of practicing nurses per 1,000 population in Korea was 6.9, which is lower than that in OECD countries, 8.8 [1]. Korean nurses, unlike those in Western countries, are usually assigned to clinical practices and rarely provide assistance with patients’ daily functions [2, 3]. As a result, informal caregivers, such as family members, generally take care of patients’ daily needs [4, 5]. The Korean hospital culture is different from that in the United States and in European countries in that caregivers often reside with patients in hospital wards, and there is no rigid hospital policy of restricting visiting hours. [6, 7]. According to the Korean Social Trends 2019 reports, the average time spent on family caregiving was 29.5 h per week, with a maximum caregiving time of 168 h per week [8]. Even though informal caregivers spend substantial time assisting their patients, the time costs of informal caregiving in Korea have not been estimated.

To relieve the burden of informal caregivers, the Korean government has implemented a comprehensive nursing service (CNS), assigning a team consisting of registered nurses and assistant personnel [2, 6] to designated hospital wards. It has been shown that patients and their family members are more satisfied with this new system [9, 10]. However, some studies have shown no statistical relevance [10] or have shown that nurses working in CNS wards experience worse job distress and higher turnover rates than those working in the general unit [2, 3]. Additionally, due to the short history of the CNS, systems assessment and evaluation of the health outcomes resulting from the CNS still need to be established [2]. As a result, the majority of elderly patients at this point still rely on informal caregivers. Therefore, it is necessary to understand the characteristics of elderly patients’ caregiver use under limited hospital resources to ensure sustainable management.

Andersen’s behavioral model, a theoretical framework encompassing individual and societal levels of healthcare service use, considers an individual’s underlying nature (predisposing factors); social influences, such as an individual’s health beliefs or health policy, that determine health service access (enabling factors); and an individual’s health status or the morbidity rate at the societal level (need factors) [11, 12]. Although numerous studies have applied this model to determine the relationship between healthcare use among elderly patients with diverse health conditions [13], the factors associated with healthcare use differed according to the socio-environmental characteristics. According to Heider’s study, elderly patients in Germany experiencing a greater level of cumulative illness had higher total healthcare costs, including inpatient treatment costs and nursing costs [14]. In addition, elderly Chinese patients who had multiple morbidities, were urban residents, and were health insurance holders were more likely to use inpatient services, showing that social contexts contributes to elderly people’s healthcare use [15].

Several studies have also examined the relationship between elderly patients’ health care costs and sociodemographic factors from multiple perspectives. In the case of elderly patients who had received acute treatment, such as abdominal surgery, intensive care to support patients’ daily functioning and strengthen their resilience to cope with stressors in the treatment process [16, 17] was needed. Additionally, informal caregivers often reduce their work hours to spend more time assisting elderly patients, which may create financial burdens [18, 19]. Joo et al. found that informal caregiving costs per stroke survivor in the United States were substantial. They emphasized that further research is needed on the potential factors affecting caregiving burden on the family [20].

However, there is limited research on the factors associated with the use of informal caregivers by Korean elderly patients and the social costs. Kim and Lee demonstrated a significant association between inpatient service use and age, marital status, and chronic disease status without a specific focus on the aged population [21]. Additionally, elderly patients with disability and low income levels without proper public assistance had fewer inpatient days and higher out-of-pocket payments, excluding formal or informal caregiving costs [22]. Additionally, hospitalized elderly patients’ gender, age, and health status were related to receiving assistance from paid caregivers [23, 24], whereas the determinants of using informal caregivers and the related costs have not yet been discussed.

Therefore, this study aimed to analyze the socioeconomic factors affecting caregiving costs among elderly patients hospitalized due to acute illness or exacerbation of chronic diseases. The specific aims are to determine the factors associated with using both formal and informal caregivers and identify factors affected by caregiving costs.

## Methods

### Data source

This study uses the 2017 Korea Health Panel Study (KHP) data for the analysis. The KHP provides information about individual healthcare behavior, health status, usage of health services, and healthcare expenditures and is jointly carried out by the Korea Institute for Health and Social Affairs and the National Health Insurance Service [4].

### Study participants

**Fig. 1:**
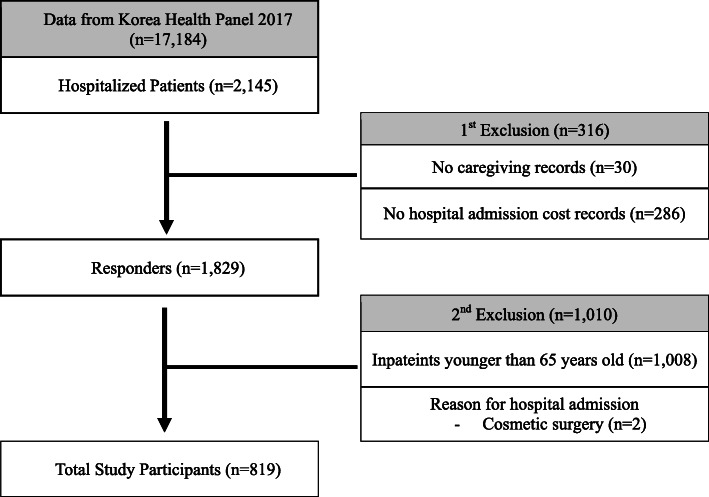
Flow diagram for the selection of study participants

A total of 2,145 individuals who had experienced hospitalization within the last year were selected from the data. Those who had no caregiving records (*n* = 30) or hospital admission cost records (*n* = 286) were excluded. Of those who had fully answered the survey items, inpatient service users younger than 65 years old (*n* = 1,008) and those who had been admitted to the hospital for cosmetic surgery (*n* = 2) were also excluded. Therefore, a total of 819 elderly inpatients aged more than 65 years were included in the analysis (Fig. 1).

### Definition of variables

#### Formal and informal caregivers

Caregiving is defined as the provision of acutely hospitalized patients with assistance to carry out daily activities by paid caregivers, voluntary organizations, or relatives. The types of caregivers were classified according to their relationship with the patients. Formal caregivers were defined as those who were financially paid for caregiving services, whereas informal caregivers were relatives or unpaid nonrelatives of the inpatients. Informal caregiving at the patient’s residence after discharge from the hospital was excluded from the cost estimation to maintain consistency, as caregiving was limited to nursing at the time of hospitalization.

#### Caregiving cost estimation

The study participants answered questions about their major caregivers during hospitalization. Formal caregiving costs were calculated by multiplying the average formal caregiver payment per day by the total number of days that caregiving services were received. The replacement cost method was used to estimate informal caregiving costs based on the assumption that hiring paid caregivers can be a substitute for engaging in informal caregiving activities [4, 20]. The reason for using the replacement cost method is that the care costs are limited to those incurred while caring for patients using healthcare services, and informal care provided during hospitalization is a suitable substitute for paid caregivers, from which follows the relevance of measuring the economic value of informal caregiving [25]. Additionally, there are limitations in applying other costing measurements, such as the opportunity cost method because most informal care providers, even if they are not engaged in economic activities, do not provide detailed information that can be used to distinguish and estimate the value of the time given up to provide actual care. Paid care services are readily apparent alternatives to the informal care provided by medical institutions during treatment, such as inpatient care, therefore, the replacement cost method is a more reasonable approach than the opportunity cost method, which requires estimating caregivers’ wage levels [4, 25]. The costs of informal caregivers’ time were estimated by applying the market price of equivalent services. The average per-day payment to paid caregivers used to estimate the cost of daily informal caregiving is based on the Healthcare Experience Survey 2018 [26]. Therefore, informal caregiving costs were estimated by multiplying the total number of days of hospitalization by the daily cost of informal caregiving.

### Independent variables

The sociodemographic characteristics of individuals from different contextual backgrounds influence healthcare service use [12]. This study applied Andersen’s behavioral model to explore possible sociodemographic factors that can explain the pattern of elderly patients’ health service utilization during their hospital admission at both the individual and societal levels. Gender, age, education level, and household type were predisposing factors. Enabling factors consisted of the household income level and the type of health insurance that inpatients held at the time of hospitalization. The type of health insurance was classified into two types: national health insurance (NHI) or the medical aid program, supporting low-income household, or patients with rare, intractable chronic disease. However, this study excluded the enrollment status of long-term care insurance (LTCI), which covers the direct healthcare expenses of elderly patients over 65 years old who obtain prior approval for services through medical assessment of their functional ability, because fewer than 1 % of participants were enrolled in this scheme. Therefore, it was concluded that LTCI would have a diminishingly small impact on the analysis. Additionally, the CNS could not be accounted for because information related to the CNS began to be collected only in 2018. Instead, survey results regarding the receipt of financial aid for hospital admission from nongovernmental sources such as nonprofit organizations or private insurance were used to measure the receipt of support for the hospital admission. Finally, the medical institutions in which patients had been primarily hospitalized were categorized into two groups: tertiary hospitals with a minimum of 100 beds providing specialized services in major medical fields and secondary hospitals with at least 30 beds. In the case of the need factors, the Charlson comorbidity index (CCI) and having a physical or psychological disability, as determined by codes derived from patients’ reports of receiving a medical diagnosis, were used to assess the need for healthcare services among inpatients. The Korean Standard Classification of Disease version 7 (KCD-7) is the Korean modification of the International Classification of Diseases and Related Health Problems (ICD-10) for international comparisons. In this study, the disease type is defined by using categories at the block level [27, 28]. The types of disease with which patients were diagnosed were classified into eight categories: cancer; diseases of the eye and adnexa; diseases of the circulatory system; diseases of the respiratory system; diseases of the digestive system; diseases of the musculoskeletal system and connective tissues; injury, poisoning and certain other consequences of external causes, and other symptoms. Additionally, this study used CCI to measure the risk of medical needs and resources required during the admission based on each patient’s comorbidity. The CCI was initially developed to predict the risk of one-year mortality but used to adjust a various range of health outcomes afterward [29, 30]. Comorbidities diagnosed by a physician before and during the hospital admission were identified using KCD-7 codes [31]. After confirming each patient’s type of comorbidities, CCI was calculated and categorized into three levels; 0, 1, and more than 2 scores. Finally, survey results regarding a physical or psychological disability were accounted for to include patients’ endogenous health issues that require essential assistance.

### Statistical analysis

The caregiving cost is the sum of payments to paid caregivers and the estimated informal caregiving costs. The distribution of caregiving costs is zero for nonusers and positive values for those receiving caregiving services. Therefore, a two-part model was used to determine the factors related to using assistance from caregivers and to estimate the incremental caregiving costs after stratifying subjects according to their age. Logistic regression analysis was used to estimate the probability of receiving caregiving in the first part. Additionally, the factors that determine caregiving costs were analyzed in the second part using a generalized linear model (GLM) with a log link and gamma distribution. The incremental effects, a combined logit and GLM version of the two-part model, on caregiving costs were presented [32, 33]. It predicted the mean caregiving cost difference in predisposing factors, enabling factors, and need factors among elderly inpatients. The variance inflation factors confirmed that there were no multicollinearity problems, and the model specification test showed that there were no misspecification errors. KHP data cross-sectional sampling with specific weights adjusting for unequal selection probability was designed to accordingly assign and estimate the structure of the Korean population from the current survey. Therefore, this study applied sampling weights to estimate nationally representative results for the proportion of the descriptive characteristics and the average healthcare expenditure, including caregiving costs, as in a previous study that used KHP data to study inpatient hospital services [34]. Stata (Stata Corp, Texas, US) version 16 was used for all statistical analyses.

## Results

### General characteristics of elderly inpatients according to the status of receiving assistance from caregivers

Table 1 shows that the average age of elderly inpatients with caregivers (76.7 ± 6.5) is older than that of those without help from caregivers (73.7 ± 6.7). Elderly inpatients with or without caregivers showed some common clinical characteristics. Elderly inpatients with caregivers (10.4 %) and without caregivers (6.8 %) had a CCI score of more than 2. Additionally, both elderly inpatients with caregivers (81.2 %) and elderly inpatients without caregivers (75.4 %) experienced financial burdens due to hospital charges. However, there were some differences between elderly inpatients with caregivers and those without caregivers. The proportion of living alone was higher among elderly inpatients without caregivers (30.0 %) than among those with caregivers (21.1 %). In addition, more than half of elderly inpatients with caregivers were receiving financial support (57.7 %) and were treated at a tertiary hospital (70.0 %). However, most elderly inpatients without caregivers were excluded from financial help (61.0 %) and admitted to a secondary hospital (52.5 %).

**Table 1:** Characteristics of elderly inpatients by the status of caregiver assistance

Variable (n, weighted %)		With Caregivers (*n*=574)	Without Caregivers (*n*=245)	*p*-value
Gender	Male	244 (43.0)	90 (37.7)	0.12
	Female	330 (57.0)	155 (62.3)	
Age (weighted mean ± SD^a^)		76.7 ± 6.5	73.7 ± 6.7	0.00^**†**^
Education Level	≤Junior high	447 (73.7)	200 (80.2)	0.22
	≥Senior high	127 (26.3)	45 (19.8)	
Household Type	With spouse	299 (49.9)	100 (40.6)	0.01^*^
	Alone	122 (21.1)	74 (30.0)	
	With spouse and another family member	105 (20.0)	42 (18.0)	
	With another family member	48 (9.0)	29 (11.4)	
Household Income	Low (1^st^)	246 (39.9)	106 (40.1)	0.95
	Low-Middle (2^nd^)	156 (26.4)	66 (26.6)	
	Middle (3^rd^)	86 (15.4)	38 (14.6)	
	Middle-High (4^th^)	45 (8.9)	21 (11.2)	
	High (5^th^)	41 (9.4)	14 (7.5)	
Type of Public Insurance	NHI	517 (89.4)	214 (88.2)	0.24
	Medicaid	57 (10.6)	31 (11.8)	
Financial Support	No	246 (42.3)	150 (61.0)	0.00^**†**^
	Yes	328 (57.7)	95 (39.0)	
Financial Burden due to Hospital Charges	Not burdensome at all ~ Somewhat manageable	104 (18.8)	57 (24.6)	0.09
	Somewhat burdensome ~ Very burdensome	470 (81.2)	188 (75.4)	
Type of Medical Institution	Secondary hospital	183 (30.0)	128 (52.5)	0.00^**†**^
	Tertiary hospital	391 (70.0)	117 (47.5)	
CCI	0	446 (77.7)	215 (86.3)	0.01^**†**^
	1	70 (11.9)	19 (6.9)	
	≥2	58 (10.4)	11 (6.8)	
Type of Diagnosed Disease at the Time of Hospital Admission	Cancer (Neoplasms)	72 (12.5)	19 (9.9)	0.00^**†**^
	Diseases of the eye and adnexa	41 (6.4)	63 (26.6)	
	Diseases of the circulatory system	81 (15.9)	14 (5.1)	
	Diseases of the respiratory system	51 (8.9)	15 (5.2)	
	Diseases of the digestive system	45 (8.7)	24 (8.7)	
	Diseases of the musculoskeletal system and connective tissue	107 (18.9)	27 (10.6)	
	Injury, poisoning and certain other consequences of external causes	84 (13.5)	35 (15.5)	
	Other symptoms and signs	93 (15.2)	48 (18.4)	
Physical/Psychological Disability	No	462 (80.0)	211 (87.3)	0.05
	Yes	112 (20.0)	34 (12.7)	

*Note.*^a^*SD* Standard Deviation

^*^p<0.05

^**†**^*p*<0.00

### Average total healthcare expenditures for hospital admission

There was a difference in healthcare expense levels among elderly inpatients according to their status of receiving aid from caregivers (Table 2). Hospital charges for inpatient services are categorized as follows; covered by NHI, paid through cost-sharing, and paid out of pocket. Elderly inpatients with caregivers paid a total of US$ 3,966 ± 5,158 for their hospital admission, while elderly inpatients without caregivers had lower total healthcare costs (US$ 1,358 ± 1,605). Additionally, elderly inpatients with caregivers spent more on hospital charges in each category than those without caregivers, showing a statistically significant difference (*p* < 0.00). The average caregiving cost of paying for formal caregivers was US$ 1,291 ± 1,436, whereas the estimated average informal caregiving cost was US$ 1,034 ± 2,283 (Table 3).

**Table 2:** Average hospital charges for inpatient care per elderly inpatient

Average of hospital charges per elderly inpatient in US$ (Weighted Mean ± SD^a^)	Elderly inpatients with caregivers (*n* = 574)	Elderly inpatients without caregivers (*n* = 245)	*p*-value
Total of inpatient service payment	3,966 ± 5,158	1,358 ± 1,605	0.00^**†**^
Covered by NHI	2,715 ± 3,938	919 ± 1,227	0.00^**†**^
Paid through cost sharing	575 ± 791	234 ± 343	0.00^**†**^
Paid out of pocket	675 ± 1,242	204 ± 369	0.00^**†**^

*Note.* All costs were adjusted to US$ (a currency exchange rate of Korean Won ₩109,950 to US dollar $100)

^a^*SD *Standard Deviation

^*^p < 0.05

^†^*p* < 0.00

**Table 3:** Average caregiving costs per elderly inpatient according to the type of caregiver

Average of caregiving costs per elderly inpatient in US$ (Weighted Mean ± SD^a^)		*p*-value
Formal caregiving costs	1,291 ± 1,436	0.33
Informal caregiving costs	1,034 ± 2,283	

*Note.* All costs were adjusted to US$ (a currency exchange rate of Korean Won ₩109,950 to US dollar $100); Informal caregiving costs were estimated by replacement cost method

^a^*SD *Standard Deviation

### Determinants of caregiver use and caregiving costs among elderly inpatients

Elderly inpatients who resided by themselves were less likely to use caregivers (OR: 0.49, *p* < 0.00) than those who lived with a spouse, but the household type had no statistically significant relationship with caregiving costs (β: 0.07, *p* = 0.42) (Table 4). However, those who had received financial support (OR: 2.68, *p* < 0.00), or used a tertiary hospital (OR: 2.77, *p* < 0.00) were more likely to use caregivers during hospitalization. Of these, elderly inpatients with financial support (β: 0.64, *p* < 0.00) spent 64 % more on caregiving costs than nonreceivers, and elderly inpatients with medicaid insurance (β: 0.54, *p* < 0.05) spent 54 % more on caregiving costs. Additionally, elderly inpatients receiving financial support spent on average US$ 627 (95 % CI: US$ 354 ~ US$ 899) more on caregiving costs than nonreceivers.

**Table 4:** Analysis of caregiver use and its costs among elderly inpatients

Variable		Logit Model (*n* = 819)	GLM Model (*n* = 574)	
		**Odds Ratio** **(95 % CI**^a^**)**	**Coefficient (95 % CI**^**a**^**)**	**Incremental Cost in US$** **(95 % CI**^**a**^**)**
Gender (Ref: Male)	Female	0.93 (0.62 ~ 1.39)	0.02 (-0.25 ~ 0.30)	4 (-205 ~ 214)
Education Level (Ref: ≥Senior High)	≤Junior High	1.42 (0.86 ~ 2.34)	-0.26 (-0.61 ~ 0.09)	− 114 (-352 ~ 125)
Household Type (Ref: With spouse)	Alone	0.49^†^ (0.31 ~ 0.77)	0.07 (-0.26 ~ 0.41)	− 88 (-313 ~ 137)
	With spouse and another family member	1.16 (0.67 ~ 2.02)	0.39 (-0.01 ~ 0.79)	360 (-72 ~ 791)
	With another family member	0.73 (0.40 ~ 1.32)	-0.01 (-0.38 ~ 0.36)	− 60 (-317 ~ 197)
Household Income (Ref: Low (1st ))	Low-Middle (2nd )	0.88 (0.57 ~ 1.36)	0.04 (-0.26 ~ 0.35)	8 (-211 ~ 227)
	Middle (3rd )	0.83 (0.46 ~ 1.52)	0.27 (-0.17 ~ 0.71)	166 (-211 ~ 542)
	Middle-High (4th )	0.55 (0.25 ~ 1.18)	-0.21 (-0.63 ~ 0.21)	− 216 (-462 ~ 30)
	High (5th )	0.84 (0.39 ~ 1.79)	0.58 (-0.34 ~ 1.52)	476 (-602 ~ 1,553)
Type of Public Insurance (Ref: NHI)	Medicaid Type 1/Type 2	1.05 (0.62 ~ 1.78)	0.54* (0.07 ~ 1.02)	498 (-84 ~ 1,079)
Financial Support (Ref: Non-Receiver)	Receiver	2.68^†^ (1.83 ~ 3.93)	0.64^†^ (0.35 ~ 0.94)	627^†^ (354 ~ 899)
CCI (Ref: 0)	1	1.03 (0.58 ~ 1.80)	-0.11 (-0.46 ~ 0.25)	-68 (-320 ~ 184)
	≥ 2	0.94 (0.40 ~ 2.16)	0.04 (-0.38 ~ 0.47)	18 (-334 ~ 370)
Physical/Psychological Disability (Ref: No)	Yes	1.53 (0.97 ~ 2.40)	-0.07 (-0.39 ~ 0.24)	26 (-215 ~ 267)
Types of Medical Institution (Ref: Secondary Hospital)	Tertiary hospital	2.77^†^ (1.91 ~ 4.01)	-0.20 (-0.46 ~ 0.06)	69 (-129 ~ 267)

*Note.* All costs were adjusted to US$ (a currency exchange rate of Korean Won ₩109,950 to US dollar $100)

^a^*CI *Confidence Interval

^*^p < 0.05

^†^*p* < 0.00

## Discussion

This study investigated that the average hospital charge for inpatient care per elderly inpatient with the caregiver was $ 3,966, which is almost three times higher than those of elderly patients without caregivers ($1,358). According to Table 1, there was a significant difference of need factor in that the proportion of CCI more than 2 for elderly inpatients with and without caregivers were 10.4 and 6.8 %, respectively. It supports previous findings from other studies in Australia [35] and in the US [36] that the higher the severity of chronic disease, the more medical resources are needed during hospitalization for acute symptoms. Consequently, this relates to causing higher financial risks. Additionally, considering that the hospital charge in Korea only accounts for treatment costs, elderly inpatients with caregivers are under a higher economic burden from hospital admission, if indirect medical expenses, such as transportation fees and caregiving costs, are included.

This study also demonstrated that financial beneficiaries and tertiary hospital patients were positively associated with caregiver use, while those living by themselves were less likely to receive assistance from caregivers. Social capital at both the individual and aggregate levels was related to caregiver use among elderly inpatients. From the individual perspective, elderly inpatients who live by themselves had 0.49 times lower odds of using caregivers during hospitalization. This finding supports Sok et al.’s finding that seniors living alone had fewer motives to engage in health-promoting behavior due to the absence of others in the family living arrangement [37]. This highlights that elderly inpatient living alone have access to limited social support in their time of need, which may lead to a considerable risk of health deterioration.

On the other hand, elderly inpatient who receive benefits from private insurance or financial subsidies from nonprofit organizations are 2.77 times more likely to use caregivers and have 64 % times of the caregiving costs. A plausible explanation for this phenomenon is that patients with a lower financial burden have higher opportunity costs during inpatient care, and therefore have more incentives to use caregivers during hospitalization. Following the study from Jeon et al., the odds of inpatient care utilization are higher among patients who hold private health insurance [38]. Additionally, a review of empirical studies showing the characteristics of private insurance holders under universal health care systems in Europe, Australia, and Israel showed that private insurance could be the influential factor for health care financing among those who can actually pay and have a higher tendency to receive more support to manage their health [39].

This study also showed that medicaid beneficiaries are 54 % times more likely to spend caregiver costs. This can be explained that medicaid beneficiaries are usually more susceptible to health risks than NHI beneficiaries due to socioeconomic disparities [40, 41]. Therefore, they may require more medical resources to manage their health during hospital admission. Additionally, medicaid beneficiaries are less likely to pay high treatment costs with a relatively lower financial burden, considering that the medicaid program is eligible for the low-income households based upon the National Basic Living Security Act [41] and gain more opportunity to use caregiver. This coincides with the results from Gong et al., who found that elderly individuals who hold health insurance are more willing to use inpatient care, giving them better access to medical services [15].

Similar to the previous context, inpatients at tertiary hospitals are 2.77 times more likely to use caregivers, showing that the influence of the healthcare system at the aggregate level could be a prime factor in determining elderly inpatients’ health behavior. Elderly inpatients from this study reported the type of hospital they had generally used within the last year, and the responses reflected that patients’ healthcare use is related to their highest expectations regarding their health management of a particular type of hospital. Patients in Korea have more access to higher hospital levels, a health behavior unique to Korea that often leads to a high concentration of patients in large hospitals [42, 43]. Therefore, patients are likely to use hospitals with highly equipped infrastructure and intensive care, based on their priority for managing their health. Along with this trend, patients’ tendency to use caregivers results from the willingness to utilize quality resources to manage their health.

In contrast to a previous study [44], this study showed no statistically significant relationship between the CCI and caregiving costs among elderly inpatients. It can be assumed that the focus of this study was on hospitalized patients with general problems rather than patients with a particular type of major chronic disease. Therefore, elderly patients with a particular type of disease or those with similar symptoms and clinical severity should be further studied to identify a detailed relationship between the comorbidity status and caregiver use.

This study also showed no statistically significant relationship between physical or psychological disability and caregiver use, with 1.53 odds. However, judging from the 95 % CI and the p-value for this association, the current status of disability is of borderline significance. Statistical significance and the width of the confidence limits are influenced by the sample size [45]. In addition, several studies discussed that elderly patient with disability continuously needed adequate interventions at the proper time in multiple dimensions due to their high dependency on caretakers [17, 46–48]. Thus, the current status of disability may be related to the caregiver use if the more sample size of patients with a disability had been analyzed.

While the present findings imply the association of certain significant social determinants with caregiver use and its expenses, they are not without several limitations. First, the functional ability level was not included in the analysis due to the limited availability of information. Although physical or psychological disability status was analyzed and exhibited a no significant relationship with caregiver use, the degree of its association may differ by the magnitude of functional ability. Another limitation is that this study focused on inpatients with various types of diseases rather than considering patients with a particular disease. As a result, cases of critical health conditions such as terminal cancer are underrepresented. Therefore, this study may not adequately reflect the specific situations of those with severe health conditions. If the duration of the disease diagnosis or the treatment process required for a particular type of disease had been given, patients’ health status could have been more precisely clarified. Last, there is a potential for recall bias that may cause the underestimation of the costs of informal caregiving, since this study used self-reported data. However, this study used data on health utilization for elderly inpatient services that were collected for one year period, which is relatively short and may enhance the accuracy of the calculated healthcare costs. Regardless of these limitations, this study indicates the importance of identifying the caregiving utilization of elderly inpatients and related expenditures while monitoring elderly patients’ relapse and their social resources, which are the focal points to relieve the caregiving burden on elderly patients’ households.

## Conclusions

The use of caregivers and the concomitant costs are associated with elderly inpatients’ social capital during acute situations. The influences of health status and social determinants differ by age group. Therefore, differential approaches to mitigating time and financial burdens should be adopted considering the age effect. Sustainable healthcare management programs that periodically examine the elderly patients’ health status and ensure the quality and assessment of social capital should be planned.

## Data Availability

The datasets used and analyzed during the current study are available in the Korea Health Panel repository, https://www.khp.re.kr:444/web/data/data.do.

## Abbreviations

CNS: Comprehensive Nursing Service
KHP: Korea Health Panel Study Data
LTIC: Long-term Care Insurance
NHI: National Health Insurance
CCI: Charlson Comorbidity Index
KCD-7: The Korean Standard Classification of Disease Version-7
ICD-10: The International Statistical Classification of Disease and Related Health Problems
GLM: Generalized Linear Model
